# Supplementary material for: High-Throughput Antibody Profiling Identifies Targets of Protective Immunity against P. falciparum Malaria in Thailand
Source: Biomolecules. 2023 Aug 18;13(8):1267. doi: 10.3390/biom13081267 (PMC10452476; doi:10.3390/biom13081267)
Supplement: Supplementary file 1 [file biomolecules-13-01267-s001.zip › Figure S1.pdf]

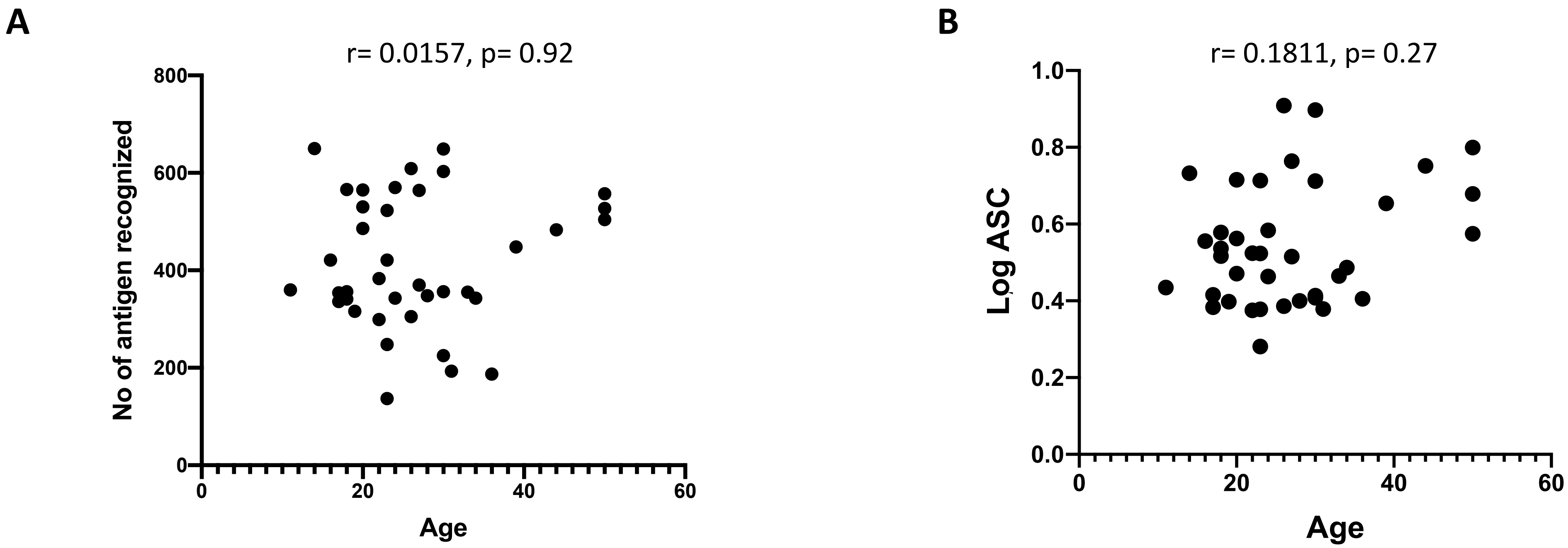

Figure S1: Comparison between the seroreactivity of antigens and the age of individuals **A**) The number of antigens recognized per individual **B**) The geometric means of log-transformed AlphaScreen count (ASC) of all antigens per individual. The correlation analysis was performed using the Spearman rank test.
