# Supplementary material for: High-Throughput Antibody Profiling Identifies Targets of Protective Immunity against P. falciparum Malaria in Thailand
Source: Biomolecules. 2023 Aug 18;13(8):1267. doi: 10.3390/biom13081267 (PMC10452476; doi:10.3390/biom13081267)
Supplement: Supplementary file 1 [file biomolecules-13-01267-s001.zip › Figure S2.pdf]

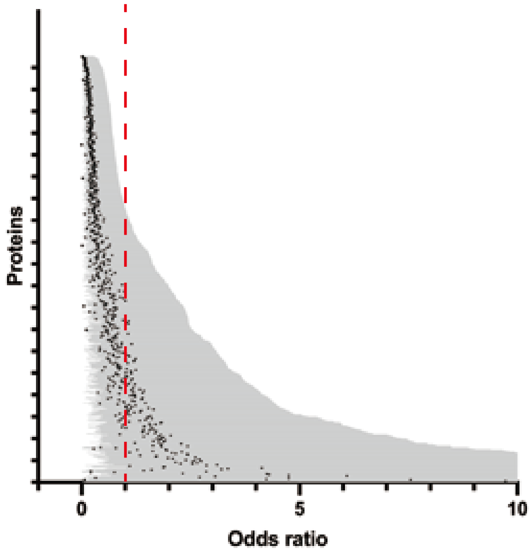

Figure S2. Comparison of the antibody responses with the risk of clinical malaria. The association between antibody responses and the risk of clinical malaria as analyzed by univariate logistic regression. Black dots indicate the odd ratios, and error bars indicate a 95% confidence interval, and the red vertical dashed line represents an odds ratio of 1. The 247 antigens exhibited a significant negative association with malaria outcomes shown in the upper section. The very high upper confidence intervals derived from 48 antigens at the bottom are truncated in the figure. The complete list of all proteins is included in Table S3.
